# Identification of Novel Broad-Spectrum Leaf Rust Resistance Sources from Khapli Wheat Landraces

**DOI:** 10.3390/plants11151965

**Published:** 2022-07-28

**Authors:** K. Raghunandan, Jatin Tanwar, Shivanagouda N. Patil, Ajay Kumar Chandra, Sandhya Tyagi, Priyanka Agarwal, Niharika Mallick, Niranjana Murukan, Jyoti Kumari, Tanmaya Kumar Sahu, Sherry R. Jacob, Atul Kumar, Suresh Yadav, Sneha Nyamgoud, Vinod, Amit Kumar Singh, Shailendra Kumar Jha

**Affiliations:** 1 Division of Genetics, ICAR-Indian Agricultural Research Institute, New Delhi 110012, India; raghunandan.plants@gmail.com (K.R.); jatintanwar969@gmail.com (J.T.); shivanagoudapatil6690@gmail.com (S.N.P.); ajaymbge29@gmail.com (A.K.C.); sandhyatyagi4@gmail.com (S.T.); priyankaagarwal11@gmail.com (P.A.); niharikamallick@gmail.com (N.M.); mniranjana2010@gmail.com (N.M.); sureshiari.genetics@gmail.com (S.Y.); nymagoudsneha15sept@gmail.com (S.N.); vinod.genetics@gmail.com (V.); 2 ICAR-National Bureau of Plant Genetic Resources, New Delhi 110012, India; jj.gene@gmail.com (J.K.); tanmayabioinfo@gmail.com (T.K.S.); sherry.jacob@icar.gov.in (S.R.J.); 3 Division of Seed Science and Technology, ICAR-Indian Agricultural Research Institute, New Delhi 110008, India; atulpathiari@gmail.com

**Keywords:** leaf rust, resistance source, khapli landrace, wheat, SNP genotyping

## Abstract

Wheat leaf rust caused by *Puccinia triticina* Eriks is an important disease that causes yield losses of up to 40% in susceptible varieties. Tetraploid emmer wheat (*T. turgidum* ssp. *Dicoccum*)*,* commonly called Khapli wheat in India, is known to have evolved from wild emmer (*Triticum turgidum* var. *dicoccoides*), and harbors a good number of leaf rust resistance genes. In the present study, we are reporting on the screening of one hundred and twenty-three dicoccum wheat germplasm accessions against the leaf rust pathotype 77-5. Among these, an average of 45.50% of the germplasms were resistant, 46.74% were susceptible, and 8.53% had mesothetic reactions. Further, selected germplasm lines with accession numbers IC138898, IC47022, IC535116, IC535133, IC535139, IC551396, and IC534144 showed high level of resistance against the eighteen prevalent pathotypes. The infection type varied from “;”, “;N”, “;N1” to “;NC”. PCR-based analysis of the resistant dicoccum lines with SSR marker *gwm508* linked to the *Lr53* gene, a leaf rust resistance gene effective against all the prevalent pathotypes of leaf rust in India and identified from a *T. turgidum* var. *dicoccoides* germplasm, indicated that *Lr53* is not present in the selected accessions. Moreover, we have also generated 35K SNP genotyping data of seven lines and the susceptible control, Mandsaur Local, to study their relationships. The GDIRT tool based on homozygous genotypic differences revealed that the seven genotypes are unique to each other and may carry different resistance genes for leaf rust.

## 1. Introduction

Wheat is one of the most widely cultivated staple food crops globally. Worldwide, nearly one-fifth of the total arable land is cultivated for wheat, with a total production of 775.7 mt (Source: USDA, 2020–21). According to the 4th Advance Estimates of production in India, wheat is grown on 31.6 million ha with production and average productivity of 109.52 mt, and 3464 kg/ha, respectively (MoA & FW, 2020–21). The crop is cultivated across the country but is mainly confined to the northern Indo-Gangetic plains, central zones, and peninsular regions (https://farmer.gov.in/M_cropstaticswheat.aspx, accessed on 17 May 2022). In particular, three species of wheat, i.e., *Triticum aestivum* (6X), *Triticum durum* (4X), and *Triticum dicoccum* (4X), are cultivated in India. *T. durum* and *T. dicoccum* cultivation is mostly restricted to central and peninsular India. Wheat is subjected to several biotic and abiotic stresses. The biotic factors affecting wheat include rusts, smuts, bunts, leaf blight, powdery mildew, and head scab. Wheat rusts are among the well-studied obligate fungal pathogens known for their historical relevance. The genus *Puccinia,* to which the wheat rust pathogens belong, is the largest genus of rust fungi, encompassing nearly 4000 species [1]. Wheat leaf rust (*Puccinia triticina* Eriks.) is among the world’s most significant diseases. The other two are stem rusts caused by *Puccinia graminis* f. sp. *tritici* Eriks & Henning and stripe rust caused by *Puccinia striiformis* West. f. sp. *tritici* Eriks. & E. Henning [2,3]. Wheat leaf rust is prevalent in all wheat-growing areas of the world and causes huge economic yield loss, which may vary from 40–50%, depending upon the stage of attack [4,5,6,7]. Long-distance dispersal of spores by air allows the newly evolved leaf rust pathogen races to spread rapidly. Although fungicides can manage leaf rust, genetic resistance within the wheat gene pool has been proven to be the most effective, economical, sustainable, environmentally-friendly and viable approach to controlling leaf rust [8]. Identification of durable and broad-spectrum resistance through genetically diverse genetic resources can enhance the durability of leaf rust resistance. In the past few decades, eighty-two genes responsible for leaf rust resistance have been identified and catalogued in wheat [9,10,11]. Only a few R-genes reported are known to control leaf rust resistance in dicoccum wheat have been identified. Among the catalogued *Lr* genes, more than 50 percent have been known to originate from wild or related species. Due to the evolution of new virulent races, several resistance genes are known to be ineffective. R genes’ effectiveness depends on the development of new pathogen strains. Due to selection pressure, the mutation of the *Avr* gene defines the ability of the pathogen to overcome resistance, leading to loss of recognition by the corresponding *R* gene [12]. Therefore, the continuing need to search for new resistance genes from different available sources is essential. The pathogen’s virulence and the prevalence of new pathotypes keep changing over time. Pathotype 77-5 (121R63-1) of *P. triticina* was the predominant pathotype for the last 20 years. Since 2016, the pathotype 77-9 (121R60-1) has become more prevalent [13]. Leaf rust resistance in Indian wheat varieties is mainly based on *Lr1, Lr3, Lr9, Lr10, Lr13, Lr14a, Lr17, Lr19, Lr22, Lr23, Lr24, Lr26,* and *Lr34* genes [14]. The gene *Lr23*, derived from the cultivar “Gaza”, has commonly been postulated in Indian durum wheat genotypes. In addition, *Lr3* [15] and *Lr14a* [16] have been reported to be present in Indian durum wheat. Although several leaf rust resistance genes have been postulated in bread wheat and durum wheat, the information regarding emmer wheat resistance is scarce. 

Depending on the species’ genomic architecture, the wheat gene pool is divided into three groups: the primary, secondary, and tertiary gene pools. Many untapped sources of valuable alien genes can be found in the secondary and tertiary gene pools, but they need cytological manipulation to transfer the desirable genes. In contrast, the primary gene pool species, such as emmer wheat and its wild form, *T. dicoccoides,* can be utilized efficiently to transfer novel genes. Genetic and morphological evidence indicates that the cultivated tetraploid *turgidum* wheat, i.e., both hulled *dicoccum* forms and free-threshing *durum* varieties, are closely related to the wild wheat that is native to the Near East and traditionally called *Triticum dicoccoides* (Korn) Aaronsohn (wild emmer wheat) [17,18,19,20,21]. Genes can be transferred directly from the primary gene pool species by crossing, homologous recombination, and the selection of desirable plants as a breeding method. The direct crosses can be made between the species in the primary gene pool with common bread wheat and by developing synthetic wheat [22,23]. 

Globally, dicoccum wheat, also known as Khapli wheat in India, is confined to a few mountains and marginal areas of Italy, Ethiopia, and India. In India, it is mainly grown in Northern Karnataka, Southern Maharashtra, and parts of Tamil Nadu, Andhra Pradesh, and coastal Gujarat. This study includes a collection of dicoccum germplasms from India. The dicoccum landraces have been grown in India from time immemorial but have never been used systematically to identify broad-spectrum resistance sources. Additionally, the landraces are being maintained in the national gene bank of India at ICAR-NBPGR, and work has already been initiated to characterize these at the morpho-physiological level. Therefore, an attempt has been made in this study to identify novel and broad-spectrum resistance sources.

## 2. Results

A set of 123 germplasm lines screened for leaf rust resistance against the pathotype 77-5 initially showed a high degree of resistance and susceptibility with infection types (ITs) ranging from ‘0;’ to ‘3+’ along with the intermediate ITs (Table 1). The susceptible control, Mandsaur Local, showed an IT of ‘3+’. The screening was carried out in two main wheat crop seasons during 2019–2020 and 2020–2021. ITs for leaf rust screened in two main wheat crop seasons were tested for homogeneity of variance across groups using Levene’s test [24]. It was observed that the *p*-value of 0.5472 was not less than the significance level of 0.05. Out of the 123 germplasm lines, 16 lines showed absolute resistance with infection type ‘0;’ in both seasons. Among the sixteen resistant germplasm lines, seven lines, with a positive control (Thatcher +*Lr53*) and negative control (Mandsaur Local), were further selected for single race testing (SRT) based on morphological characteristics and ITs. 

The seven germplasm lines that showed a high resistance had ITs ranging from ‘0;’ to ‘1’, whereas the susceptible germplasm line had ITs ranging from ‘3’ to ‘3+’ in Mandsaur Local when screened against eighteen pathotypes from six diverse groups, i.e., 12, 77, 104, 107, 108, and 162 (Table 2).

**Figure 1:**
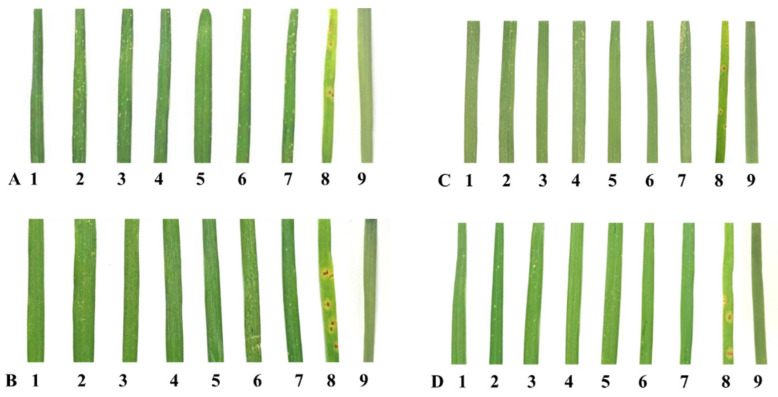
Infection type in seven dicoccum germplasm lines along with Mandsaur Local, a susceptible landrace, and Thatcher + *Lr53*, a resistant control, against four pathotypes. (**A**) 12-4, (**B**) 77-1, (**C**) 107, (**D**) 108; A representative sample is taken from each group of pathotypes. Germplasm lines (Left to Right), 1. IC138898; 2. IC47022; 3. IC535116; 4. IC535133; 5. IC535139; 6. IC 551396; 7. IC534144; 8. Mandsaur Local; 9. Thatcher + *Lr53*.

**Figure 2:**
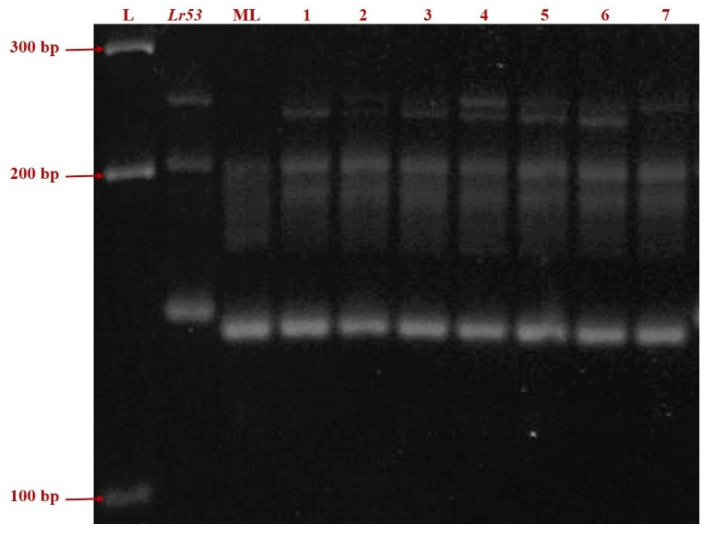
PCR amplification of the SSR marker *gwm508* in *Lr53* genetic stock, negative control ML and seven germplasm lines (Left to Right: L = ladder; Thatcher + *Lr53* = positive control; ML = Mandsaur Local = negative control; 1. IC138898; 2. IC47022; 3. IC535116; 4. IC535133; 5. IC535139; 6. IC 551396; 7. IC534144).

Infection type for leaf rust, screened against 18 pathotypes in four environments, was tested for homogeneity of variance across groups using Levene’s test. The germplasms IC138898, IC47022, IC535116, IC535133, IC535139, IC 551396, IC534144, negative control Mandsaur Local, and positive control Thatcher + *Lr53* showed *p*-values of 0.87, 0.49, 0.74, 0.37, 0.35, 0.77, 0.37, 0.46, and nil respectively. A pictorial presentation of the representative samples of ITs for each pathotype is given in Figure 1. SSR marker *gwm508* amplified a fragment length of approximately 135 bp in the *Lr53* positive control and 125 bp in the negative control, Mandsaur Local. The seven germplasm lines showed a band length of 125 bp, the same as in the negative control, Mandsaur Local (Figure 2).

**Figure 3:**
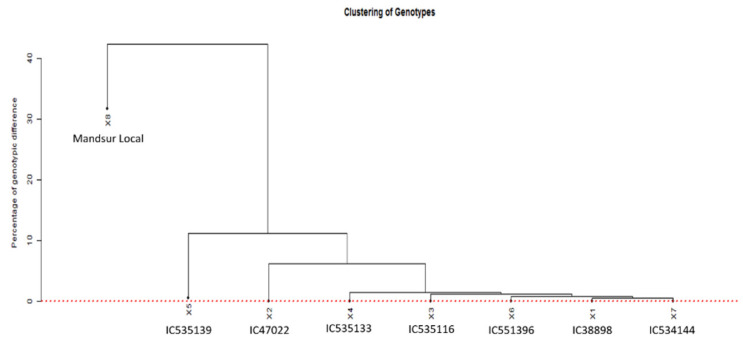
The relationship among the germplasms using the homozygous genotypic difference, computed based on identity-by-state analysis (1X = C138898, 2X = IC47022, 3X = IC535116, 4X = IC535133, 5X = IC535139, 6X = IC551396, 7X = IC534144, and 8X = Mandsaur Local).

The SNP genotyping data for the seven dicoccum germplasm lines and a durum landrace were used to verify the presence of duplicates among the selected germplasms. This was achieved using GDIRT at a 0.05% homozygous genotypic difference [25]. The GDIRT also generated a dendrogram (Figure 3) showing the relationship among the germplasms using the homozygous genotypic difference, computed based on identity-by-state analysis. The dotted red line in the figure shows the threshold homozygous genotypic difference, above which the putative ancestral nodes of all genotypes lie. It indicates the uniqueness of all the eight genotypes, though some genotypes are observed to be close to others. 

## 3. Discussion

*T. dicoccoides*, the ancestor of modern tetraploid and hexaploid wheat, and *T. dicoccum* are valuable sources of novel genetic variation for disease-resistance genes. *T. dicoccum* is a member of the primary gene pool for the hexaploid bread wheat [26]. Traits such as resistance to leaf rust disease can be transferred quickly by the conventional method of crossing and selecting desirable plants. Most of the genes for leaf rust resistance have originated from bread wheat and its wild relatives, whereas very few genes have originated from durum wheat, viz. *Lr3, Lr14a, Lr27 + Lr31, Lr61, Lr72, Lr79* and *LrCamayo* [15,16,27,28,29]. The leaf rust resistance gene *Lr36* [30] and the stripe rust resistance gene *Yr36* [31], located on 6BS, were transferred from *T*. *dicoccoides* to bread wheat. Two seedling stage leaf rust resistance genes, *Lr53* and *Yr35,* were mapped to the short arm of chromosome 6B by using the Chinese Spring monosomic series and telosomic stocks for 6B. Both were linked and transferred from *T*. *dicoccoides* to hexaploid wheat by Marais et al. (2005) [32]. *Lr14a* is a gene of dicoccum source *T. turgidum* ssp. *dicoccum* cv. ‘Yaroslav’.

**Figure 4:**
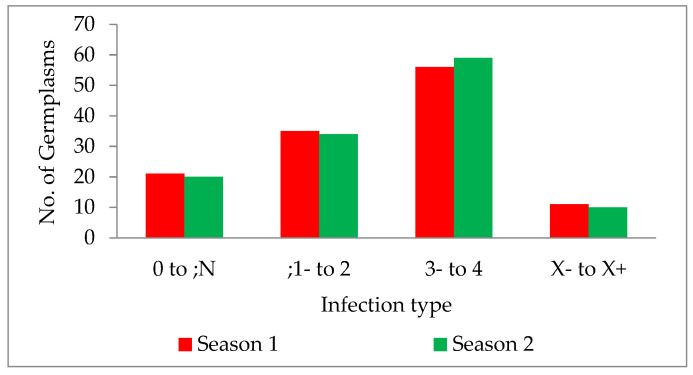
Bar graph description of germplasm infection types in two seasons, 2019–2020 and 2020–2021.

The wild form of *T. dicoccoides* and its cultivated species, *T. turgidum dicoccum*, harbour many resistance genes. The genotypes used in the current study mainly comprised the indigenous collection of dicoccum germplasm accessions from different geographical locations in India. Infection type for leaf rust, screened for pathotype 77-5 in two main wheat crop seasons, was tested for homogeneity of variance across groups using Levene’s test. The study revealed that the *p*-value of 0.5472 is not less than the significance level of 0.05. Therefore, statistically, there is no evidence to suggest that the variance in the score is significantly different for the two seasons. The germplasm lines showed a wide range of disease scores ranging from ‘0;’ (considered immune without any/with little sign of initial hypersensitive reaction) to ‘4’ (is highly susceptible; uredia profusely sporulating). The infection types observed in the collection were classified further into categories of resistant, moderately resistant, susceptible and mesothetic groups. It was observed that 17.07% and 16.26% of the total germplasm collection showed highly resistant reactions, i.e., “0” to “;N” in two consecutive seasons, respectively. This portion of resistant genotypes that show a high degree of resistance and immunity, form an integral part of the germplasm collection available, and can be further explored for identifying novel genes. Since fewer leaf rust resistance genes are identified in dicoccum, the indigenous collection with resistance becomes a source for novel resistance. A total of 28.45% and 27.64% of the germplasm collection showed resistant reactions with infection type “;1” to “2” in the two consecutive seasons, respectively (Figure 4). Aoun et al. 2016 [33] reported that the percentage of accessions with resistance to durum-specific races at the seedling stage was low, with resistance within the 496 accessions to BBBQD (ND) and BBBQD(CDL) races of 4.91% (24 accessions), and 12.27% (60 accessions), respectively. The number of germplasms with a susceptible reaction was very high, with 45.52% in season one and 47.96% in the next season. It indicates that half of the germplasm collection does not carry desirable resistance to leaf rust disease. However, these susceptible lines can be screened for stripe and stem rust to identify resistance.

Along with the above infection types, the X-type (mesothetic reaction) was observed with 8.94% and 8.13% of the entire collection in both seasons, respectively. Although an X-type reaction is considered resistant, the phenotyping of segregating lines is complex and is variable depending on the environment and parent background, making it challenging to identify the genomic location and map the genes. Minor variations observed in the infection types during the study are mainly due to the smaller variation in temperature and relative humidity during disease establishment. Many known leaf rust resistance genes are highly influenced by the environment and are temperature sensitive. The environment, particularly temperature, directly affects the expression of *R* genes [34,35]. Out of 123 germplasm lines, 16 showed high resistance with an IT fleck in both seasons consistently, without any variation in their reaction. 

Seven of the sixteen germplasm lines showing clean resistance were further selected for single-race screening. The germplasm lines with accession numbers IC138898, IC47022, IC535116, 535133, IC535139, 551396, and IC534144 showed high level of resistance against all the eighteen pathotypes. The IT varied from “;”, “;N”, “;N1” to “;NC”. Only a few resistance genes from dicoccum wheat are known to have this level of resistance against many diverse leaf rust pathotypes. 

The rust resistance gene *Lr53* reported on chromosome 6B transferred to common wheat from *Triticum dicoccoides*. The microsatellite marker *gwm508* is located on the short arm of chromosome 6B and mapped at approximately 4.5 cm, proximal to *Lr53* [36]. The germplasm lines in our study are of dicoccum species that are known to be evolved from the *T. dicoccoides.* In order to check whether the source of resistance in the seven selected germplasm lines was because of *Lr53* or not, the SSR marker *gwm508* was used to check the presence of the gene *Lr53*. The SSR marker *gwm508* has an amplicon size of 135 bp, which was observed in the positive control *Lr53* in the present study. The negative control Mandsaur Local amplified an amplicon size of 125 bp. The amplicon size obtained in the seven germplasm lines under investigation was 125 bp. Considering the above results, the gene *Lr53* may not present in the germplasm lines. 

The SNP genotyping data for the eight dicoccum germplasm lines was used to verify the presence of duplicates among the germplasms by the GDIRT tool based on homozygous genotypic differences, revealing that the seven genotypes found to be resistant against all eighteen pathotypes are different from each other. The dendrogram showing the relationship among the germplasms using the homozygous genotypic difference, computed based on identity-by-state analysis, concludes that since all the nodes fall above the red line, they are not duplicates. Although the germplasms IC138898, IC535116, IC535133, IC551396, and IC534144 seem closer to each other, still they are unique and different. The Mandsaur Local is a durum wheat cultivar separate from dicoccum wheat with a minimum of 20% difference to the nearest dicoccum germplasm IC535139 and 24% difference from the germplasm IC47022. Since the germplasms were observed to be notably different from each other, the seven germplasm lines identified in the study are highly effective against diverse pathotypes. They can be used in further studies to identify potentially novel resistance genes.

## 4. Materials and Methods

### 4.1. Plant Materials

The materials used in the current study consisted of 123 dicoccum wheat germplasm lines from indigenous collections. The collections covered the state of Karnataka, Andhra Pradesh, Madhya Pradesh, Jammu & Kashmir, Haryana, Punjab, Uttar Pradesh, Tamil Nadu, and unknown sources. The germplasm accessions were received from ICAR- National Bureau of Plant Genetic Resources, New Delhi.

### 4.2. Pathogen Used 

The pure inoculum of 19 leaf rust pathotypes (Table 3) was obtained from the Indian Institute of Wheat and Barley Research, Regional Station, Flowerdale, Shimla. The pathotypes belonged to seven groups of leaf rust pathogen: four from group 12, nine from the most virulent and prevalent pathotype group 77, two from group 104 and group 162, and one each from groups 107 and 108. The virulence and avirulance profile of the pathotypes used in the study is given in the Table S1.

### 4.3. SSR Markers and SNP Genotyping

As reported earlier, the SSR marker *gwm508* linked to the gene *Lr53* [36] was used to validate the likely presence of *Lr53* in the selected dicoccum lines. In addition, seven resistant dicoccum germplasm accessions and one durum landrace, Mandsaur Local, were genotyped for SNP using the Affymetrix 35K Wheat Breeders’ Axiom^®^ array to rule out the duplication of samples among the selected most resistant lines [37].

### 4.4. Pathotype Multiplication and Screening for Leaf Rust Resistance

All pathotypes’ initial inoculum was multiplied on the susceptible cultivar Mandsaur Local in a glasshouse. The multiplication of leaf rust pathotypes was done according to the procedure outlined in previous study [38]. 

A set of 123 germplasm accessions were screened for leaf rust resistance along with the susceptible control Mandsaur Local at the seedling stage, against the pathotype 77-5. Seedlings approximately 10 days old were inoculated by spraying them with a suspension of uredospores in water prepared with a drop of Tween-20. Inoculated seedlings were incubated in a humid chamber for 48 h. After incubation, the seedlings were kept on benches in the glasshouse at temperatures ranging between 16 °C and 24 °C, under ambient light and relative humidity conditions. Individual seedlings were scored for ITs after 12 days of inoculation following a 0–4 scale as described by Stakman et al. (1962) [39].

Out of the 123 germplasm lines, 16 germplasm lines were further selected for the study. Finally, seven germplasm lines were selected based on IT and morphological data. These seven germplasm lines, along with Thatcher + *Lr53* stock as the positive control and Mandsaur Local as the negative control, were used for single race testing (SRT). A total of 18 pathotypes were used for SRT for leaf rust resistance in isolation, using the method mentioned above for screening. Individual seedlings were scored for ITs after 12 days of inoculation, following a 0–4 scale [39]. The SRT was conducted for four seasons, where two seasons included seedling stage screening of the germplasms in the glasshouse condition in the year 2020–2021 and 2021–2022, and the other two seasons included screening in the national phytotron facility at ICAR-IARI, New Delhi in the years 202–2021 and 2021–2022.

### 4.5. DNA Extraction, Primers, and Polymerase Chain Reaction (PCR)

DNA was extracted from 15-day-old seedlings using the CTAB method [40]. DNA was quantified on 0.8% (*w/v*) agarose gel using lambda uncut DNA as standard and confirmed with a NanoDrop^TM^ Lite spectrophotometer (Thermo Fisher scientific Inc., Waltham, MA, USA). DNA samples were diluted to the working concentration of 25 ng/μL and stored at −20 °C. The SSR marker *gwm508* was reported and validated to identify the *Lr53* gene from *Triticum dicoccoides*, and was used to check the likely presence of the *Lr53* gene in the selected broad-spectrum resistant dicoccum germplasm lines. The PCR reactions were performed according to the profile described in [36]. The PCR products were separated on 4% MetaPhor gels at 80 volts for 150 min.

### 4.6. Confirmation of Uniqueness of Dicoccum Lines

To confirm that the selected seven landraces were not duplicates, the SNP genotyping data in HapMap format was subjected to the Germplasm Duplicate Identification and Removal Tool (GDIRT) [41], developed and hosted (http://webtools.nbpgr.ernet.in/gdirt, accessed on 10 April 2022) at ICAR-National Bureau of Plant Genetic Resources, New Delhi. The GDIRT identifies duplicates based on homozygous genotypic differences derived from identity-by-state analysis. From the initial set of 35,143 markers, 23,080 were retained for duplicate identification analysis after removing D genome markers, monomorphic SNPs, markers with MAF < 5%, and markers with missing data >5%.

## 5. Conclusions

Most dicoccum germplasms are yet to be explored as different biotic stress-resistant sources. Very few leaf rust resistant genes have been identified and catalogued from dicoccoides-originated wheat. Based on the mean value, 44.71% of the total germplasm accessions used in the study were resistant to pathotype 77-5. The seven selected lines for further screening showed apparent resistance to 18 pathotypes belonging to six diverse groups. The use of the *gwm508* marker, linked to the *Lr53* gene to check its likely presence in the seven germplasm lines, indicated the absence of the gene in the selected lines. The germplasms being unique, provide a diverse source of resistance to leaf rust. Hence, the selected germplasm lines can be a novel source of resistance to leaf rust and, further, can be explored to identify and map new broad-spectrum *R* genes.

## Figures and Tables

**Table 1:** Screening of dicoccum landraces for leaf rust at seedling stage against pathotype 77-5 of *Puccinia triticina* Eriks in two crop seasons (2019–2020 and 2020–2021).

S. No.	Landrace	S_1_	S_2_	S. No.	Landrace	S_1_	S_2_	S. No.	Landrace	S_1_	S_2_
1	IC535302	3	3+	42	IC535123	3	3	83	IC118763	3	3
2	IC138898	;	;	43	IC535125	X	X−	84	IC535071	X	X
3	IC118774	3	3	44	IC535131	3	3	85	IC535076	;1	;1−
4	IC531559	2	2C	45	IC535133	;1	;N	86	IC535078	3	3
5	IC535301	3	3+	46	IC535134	3	3	87	IC535083	;	;
6	IC535118	3	3	47	IC535136	;1	;1−	88	IC535085	3	3
7	IC47048	;	;	48	IC535137	;1	;1	89	IC535086	;	;
8	IC277713	3	3−	49	IC535138	3	3	90	IC535088	;1	;1−
9	IC47800	;1	;1	50	IC535139	;	;	91	IC535090	;	;1−
10	IC531969	;	;	51	IC535140	;1	;1−	92	IC535093	2	3
11	IC47022B	;	;1−	52	IC535141	3	3	93	IC535097	3	3
12	IC138418	3	3	53	IC535142	3++	3+	94	IC535105	;	;
13	IC448026	3	3	54	IC128392	3	3	95	IC535106	3	3
14	IC32513	3	3	55	IC128425	3+	3	96	IC535108	3	3
15	IC35093	3++	3+	56	IC138331	3	3+	97	IC535112	X	X
16	IC35097	3	3	57	IC138371	3+	3	98	IC535124	3	3
17	IC35119	3	3	58	IC138450	3	3	99	IC535126	2	3
18	IC35170	3	3	59	IC138455	4	3+	100	IC535129	;1	;1
19	IC35171	3	3	60	IC533783	4	3	101	IC535130	;	;
20	IC35174	3	3	61	IC534012	3	3	102	IC535143	3	3
21	IC47021	;1	;1−	62	IC534016	3	3	103	IC535144	;	;
22	IC47026	3	3	63	IC534018	3+	3	104	IC535148	3	3
23	IC47034	3	3	64	IC534586	;1	;1	105	IC535153	2	3
24	IC47035	;1	;1+	65	IC534587	X	X	106	IC138471	1+	;1
25	IC47037	X	X+	66	IC534621	3	3	107	IC138475	;1	;1
26	IC47049	;N	;	67	IC138896-A	3	3	108	IC138897	3	3
27	IC47545	4	3	68	IC138900	;1−	;1	109	IC534960	3++	3+
28	IC47548	3	3	69	IC252486	;N1	;1N	110	IC212165	;1	;1−
29	IC535304	3	3	70	IC252503	;1	;1−	111	IC212168	;1−	;1
30	IC32502	X-	X-	71	IC252504	;1	;1+	112	IC402045	0;	;
31	IC47040	;N	;N	72	IC416358	;	;	113	IC551396	;1−	;1−
32	IC112083	3	3	73	IC539302	;	;	114	IC551397	;	;
33	IC535070	4	3+	74	IC138472	3+	3	115	IC551398	;	;
34	IC535079	;1−	;1	75	IC138474	;1	;1−	116	IC551399	3	3
35	IC535081	;1N	;1N	76	IC530555	;1−	;1	117	IC551400	;	;
36	IC535082	X	X-	77	IC547564	;	;	118	IC113725	X	X-
37	IC535092	;1	;1	78	IC566241	;	;	119	IC593664	;1	;1−
38	IC535113	;1+	;1	79	IC35091	3	3	120	IC402012	X+	X
39	IC535116	;1−	;1	80	IC78699	3	3	121	IC402018	3	3
40	IC535117	;1	;1	81	IC78706	;1	;1−	122	IC402020	3	3
41	IC535120	X	X+	82	IC118727	;1	;1−	123	IC584049	;1−	;1

Note: S_1_ = Season 1 and S_2_ = Season 2.

**Table 2:** Screening of dicoccum germplasm lines along with positive control, Thatcher + *Lr53* stock, and negative control, Mandsaur Local, against 18 pathotypes of *Puccinia triticina* Eriks in four environments, at seedling stage.

S. No.	Landrace	S. No.	1.	2.	3.	4.	5.	6.	7.	8.	9.	10.	11.	12.	13.	14.	15.	16.	17.	18.
		Pathotype	12_3	12_4	12_5	12_9	77-1	77-2	77-3	77-4	77-6	77-9	77-10	77A-1	104	104-2	107-1	108	162	162-1
1.	IC138898	Y1	;	;	;	;	;	;NC	;N1	;	;N	;	;	;	;	;	;N1	;	;1−	;
		Y2	;	;	;	;	;	;N	;N1−	;	;N1−	;	;	;	;	;	;N1−	;	;1	;
		GH1	;	;	;	;	;	;N	;N	;	;N1	;	;	;	;1−	;	;N	;	;1	;
		GH2	;	;	;	;	;	;N	;N1	;N	;N	;	;1−	;	;	;	;N1−	;	;1	;
2.	IC47022	Y1	;	;	;	;	;	;NC	;N1	;	;N	;	;	;N	;	;	;N1	;	;	;
		Y2	;	;	;	;	;	;NC	;N1−	;	;N	;	;	;N	;	;	;N1−	;	;	;
		GH1	;	;	;	;1−	;	;N	;N1	;	;N1	;	;	;	;1−	;	;N1	;	;1	;
		GH2	;	;1−	;	;	;	;N1	;N	;	;N	;	;	;N	;1	;	;N1−	;	;1−	;
3.	IC535116	Y1	;	;	;	;	;N	;NC	;1−	;	;	;	;	;	;N1	;	;	;	;1−	;N
		Y2	;	;	;	;	;	;NC	;1	;	;	;	;	;	;N1	;	;	;	;1	;N
		GH1	;1−	;	;	;	;	;N	;N	;	;N1	;	;	;	;1−	;	;N	;	;1	;
		GH2	;	;	;	;	;1−	;N	;N1	;N	;N	;	;1−	;	;	;	;N1	;	;1−	;
4.	IC535133	Y1	;1−	;	;	;	;	;NC	;	;	;	;	;	;	;	;	;N1	;	;	;
		Y2	;1	;	;	;	;	;NC	;	;	;	;	;	;	;	;	;N1−	;	;	;
		GH1	;	;	;	;	;	;N	;N	;	;N1	;	;	;	;1−	;	;N	;	;1	;
		GH2	;	;	;	;	;	;N	;N1	;N	;N	;	;1−	;	;	;	;N1−	;	;1−	;
5.	IC535139	Y1	;	;	;	;	;	;N	; N	;	;	;	;	;	;	;	;	;	;N	;
		Y2	;	;	;	;	;	;N	;N	;	;	;	;	;	;	;	;	;	;	;
		GH1	;	;	;	;	;	;N	;N	;	;N1	;	;	;	;1−	;	;N	;	;	;
		GH2	;1−	;	;	;	;	;N	;N1−	;N	;N1−	;	;	;	;	;	;	;	;1−	;
6.	IC551396	Y1	;	;N	;	;	;N	;N1−	;	;	;	;	;	;	;	;	;	;	;N	;
		Y2	;	;N	;	;	;N	;N1	;	;	;	;	;	;	;	;	;	;	;N	;
		GH1	;	;	;	;	;	;N1−	;N	;	;N	;	;	;	;	;	;N	;	;1	;
		GH2	;	;	;	;	;	;N	;N1	;	;N	;	;	;	;	;	;N	;	;1−	;
7.	IC534144	Y1	;	;N	;	;	;	;NC	;	;	;	;	;1	;	;	;	;N1−	;	;	;
		Y2	;	;N	;	;	;	;NC	;	;	;	;	;	;	;	;	;N1−	;	;	;
		GH1	;	;	;	;	;	;N	;N1−	;	;N1	;	;	;	;1−	;	;N	;	;1	;
		GH2	;	;	;	;	;	;N	;N1	;N	;N	;	;1	;	;	;	;N1	;	;	;
8.	Mandsaur Local	Y1	3	3+	3	3	3	3+	3	3	3+	3+	3	3	3	3	3+	3+	3+	3
		Y2	3+	3	3	3	3+	3	3	3+	3	3	3+	3	3	3+	3	3+	3+	3+
		GH1	3+	3+	3	3	3+	3	3	3+	3	3	3+	3	3+	3	3+	3	3+	3
		GH2	3+	3	3	3+	3	3	3+	3	3+	3	3+	3	3+	3	3	3	3	3
9.	Thatcher + *Lr53*	Y1	;	;	;	;	;	;	;	;	;	;	;	;	;	;	;	;	;	;
		Y2	;	;	;	;	;	;	;	;	;	;	;	;	;	;	;	;	;	;
		GH1	;	;	;	;	;	;	;	;	;	;	;	;	;	;	;	;	;	;
		GH2	;	;	;	;	;	;	;	;	;	;	;	;	;	;	;	;	;	;

Note: Y1 = 2020–2021, Y2 = 2021–2022 main season, GH1 and GH2 = the year 2020–2021 and 2021–2022 at National Phytotron Facility, ICAR-IARI, New Delhi.

**Table 3:** List of leaf rust pathotypes used for screening diverse dicoccum wheat germplasm.

Sl. No	Pathotype Name	Sl. No.	Pathotype Name
1.	12-3	11	77-9
2.	12-4	12	77-10
3.	12-5	13	77A-1
4.	12-9	14	104
5.	77-1	15	104-2
6.	77-2	16	107-1
7.	77-3	17	108
8.	77-4	18	162
9.	77-5	19	162-1
10.	77-6	-	-

## Data Availability

The data presented in this study are available on request from the corresponding author.

## Supplementary Materials

The following supporting information can be downloaded at: https://www.mdpi.com/article/10.3390/plants11151965/s1, Table S1: Binomial designation and avirulence/virulence formula of pathotypes of leaf rust (*P. triticina*) used in the study.
