# Supplementary material for: Identification of Novel Broad-Spectrum Leaf Rust Resistance Sources from Khapli Wheat Landraces
Source: Plants (Basel). 2022 Jul 28;11(15):1965. doi: 10.3390/plants11151965 (PMC9370231; doi:10.3390/plants11151965)
Supplement: Supplementary file 1 [file plants-11-01965-s001.zip › plants-1754106-supplementary.pdf]

Supplementary Material

Table S1. Binomial designation and avirulence/virulence formula of pathotypes of leaf rust (*P. triticina*) used in the study.

S. No.	Pathotypes Designation		Avirulence/ Virulence formula
	Old	New	
1.	12-3	49R37	***Lr1,2a, 9,10,13,18,19,20,23,24,28*** / *Lr2c,3a,14a,15,17,26*
2.	12-4	69R13	***Lr1,2a, 9,13,15,17,19,23,24,26,28*** / *Lr2c,3a,10,14a,18,20*
3.	12-5	29R45	***Lr1,2a, 9,10,15,19,23,24,28*** / *Lr2c,3a,13,14a,17,18,20,26*
4.	12-9	93R37	***Lr1,2a, 9,15,19,20,23,24,28*** / *Lr2c,3a,10,13,14a,17,18,26*
5.	77-1	109R63	***Lr9,17,19,23,24,28*** / *Lr1,2a,2c,3a,10,13,14a,15,18,20,26*
6.	77-2	109R31-1	***Lr9,17,19,24,26,28*** / *Lr1,2a,2c,3a,10,13,14a,15,18,20,23*
7.	77-3	125R55	***Lr9,19,20,23,24,28*** / *Lr1,2a,2c,3a,10,13,14a,15,17,18,26*
8.	77-4	125R23-1	***Lr9,19,20,24,26,28*** / *Lr1,2a,2c,3a,10,13,14a,15,17,18,23*
9.	77-5	121R63-1	***Lr9,18,19,24,28*** / *Lr1,2a,2c,3a,10,13,14a,15,17,20,23,26*
10.	77-6	121R55-1	***Lr9,18,19,20,24,28*** / *Lr1,2a,2c,3a,10,13,14a,15,17,23,26*
11.	77-9	121R60-1	***Lr2a,2c,9,18,19,24,28*** / *Lr1,3a,10,13,14a,15,17,20,23,26*
12.	77-10	377R60-1	***Lr2a,2c, 9,18,19,24/*** *Lr1,3a,10,13,14a,15,17,20,23,26,28*
13.	77A-1	109R23	***Lr9,17,19,20,23,24,26,28*** / *Lr1,2a,2c,3a,10,13,14a,15,18*
14.	104	17R23	***Lr9,10,13,15,18,19,20,23,24,26,28*** / *Lr1,2a,2c,3a,14a,17*
15.	104-2	21R55	***Lr9,10,13,15,19,20,23,24,28*** / *Lr1,2a,2c,3a,14a,17,18,26*
16.	107-1	45R35	***Lr1,3a,9,10,17,19,20,23,24,28*** / *Lr2a,2c,13,14a,15,18,26*
17.	108	13R27	***Lr3a,9,10,15,17,19,23,24,26,28*** / *Lr1,2a,2c,13,14a,18,20*
18.	162	93R7	***Lr1,9,15,19,20,23,24,26,28*** / *Lr2a,2c,3a,10,13,14a,17,18*
19.	162-1	93R47	***Lr1,9,15,19,23,24,28*** / *Lr2a,2c,3a,10,13,14a,17,18,20,26*
